# Sexual selection on song and cuticular hydrocarbons in two distinct populations of *Drosophila montana*

**DOI:** 10.1002/ece3.75

**Published:** 2012-01

**Authors:** Paris Veltsos, Claude Wicker-Thomas, Roger K Butlin, Anneli Hoikkala, Michael G Ritchie

**Affiliations:** 1 School of Biology, University of St Andrews, Dyers Brae HouseSt Andrews, Fife, KY16 9TH, United Kingdom; 2 UPR 9034 Laboratoire Evolution, Génomes et Spéciation (LEGS), CNRS, Avenue de la TerrasseBât. 13, Jpg sur Yvette Cedex, 91198, France; 3 Animal & Plant Sciences, University of Sheffield, Alfred Denny BuildingSheffield, S10 2TN, UK; 4 Department of Biological and Environmental Science, University of JyvãskylãSurvontie 9, Jyvãskylã, 40014, Finland

**Keywords:** Courtship song, cuticular hydrocarbons, *Drosophila montana*, selection analysis, sexual selection

## Abstract

Sexual selection has the potential to contribute to population divergence and speciation. Most studies of sexual selection in *Drosophila* have concentrated on a single signaling modality, usually either courtship song or cuticular hydrocarbons (CHCs), which can act as contact pheromones. We have examined the relationship between both signal types and reproductive success using F_1–3_ offspring of wild-collected flies, raised in the lab. We used two populations of the Holarctic species *Drosophila montana* that represent different phylogeographic clades that have been separate for ca. 0.5 million years (MY), and differ to some extent in both traits. Here, we characterize the nature and identify the targets of sexual selection on song, CHCs, and both traits combined within the populations. Three measures of courtship outcome were used as fitness proxies. They were the probability of mating, mating latency, and the production of rejection song by females, and showed patterns of association with different traits that included both linear and quadratic selection. Courtship song predicted courtship outcome better than CHCs and the signal modalities acted in an additive rather than synergistic manner. Selection was generally consistent in direction and strength between the two populations and favored males that sang more vigorously. Sexual selection differed in the extent, strength, and nature on some of the traits between populations. However, the differences in the directionality of selection detected were not a good predictor of population differences. In addition, a character previously shown to be important for species recognition, interpulse interval, was found to be under sexual selection. Our results highlight the complexity of understanding the relationship between within-population sexual selection and population differences. Sexual selection alone cannot predict differences between populations.

## Introduction

Sexual selection arises due to variation in mating success between individuals in a population, and is thought to lie behind many sexually dimorphic morphological and behavioral traits. Sexual selection can lead to very rapid evolution of traits if they are unconstrained by natural selection or pleiotropic effects (Fisher 1930; Lande 1981). The possibility of rapid divergence due to sexual selection has sparked interest in its potential to generate reproductive isolation between populations that may contribute to speciation (Gavrilets 2000; Panhuis et al. 2001; Ritchie 2007; Kraaijeveld et al. 2010). Sexual selection could act alone on mating behavior, generating sexual isolation, or it could act in concert with ecological selection, if mating traits indicate ecological adaptation, and accentuate reproductive isolation (Dieckmann and Doebeli 1999; van Doorn et al. 2009; Weissing et al. 2011). Many models emphasize the interaction between sexual and ecological selection in sympatry, but both processes could also contribute to allopatric divergence.

The regression-based method of selection analysis introduced by Lande and Arnold (1983) allows the identification of traits under sexual selection and the estimation of its strength (Lande and Arnold 1983; Brodie et al. 1995; Kingsolver et al. 2001; Blows 2007). The correlative nature of selection analysis is counterbalanced by its ability to capture biologically realistic information by taking into account variation in multiple variables. It is particularly useful to study animal communication because of its multivariate nature. To better understand the involvement of sexual selection in population differentiation, we need more comparative studies that jointly analyze variation among populations in multiple traits and sexual selection on those traits within different, distinct populations. Few such studies have been conducted but those that have suggest that large differences are possible among populations in the direction and/or strength of selection (e.g. Gosden and Svensson 2008; Rundle et al. 2008; Cornwallis and Uller 2010).

*Drosophila* provides many opportunities for the study of sexual communication. Acoustic communication in *Drosophila* usually involves “song” produced by males vibrating their wings to generate near-field acoustic signals during courtship (Ewing and Bennet–Clark 1968; Schilcher 1976). *Drosophila* song is usually species specific (Ritchie et al. 1999) and responds rapidly to both artificial and sexual selection in the laboratory (Ritchie and Kyriacou 1996; Snook et al. 2005). For some *Drosophila* species, the role of contact pheromones is played by cuticular hydrocarbons (CHCs), sensed by either gustatory or olfactory receptors (Ferveur 2005; Nakagawa et al. 2005). Typically, a *Drosophila* CHC profile is made up of about 30 long-chain hydrocarbons (Howard 1993; Jallon and Wicker–Thomas 2003). The compounds can be species-, sex-, or developmental stage specific and are known to contribute to sexual isolation between some species (Howard et al. 2003; Ferveur 2005; Smadja and Butlin 2009). There are fewer studies of their role in sexual selection, especially involving both song and CHCs (e.g., Etges et al. 2009), which is particularly important if the signal modalities interact (Rybak et al. 2002). CHCs may also function in environmental adaptation; for example, there is evidence for a role in desiccation resistance and starvation tolerance in *Drosophila melanogaster* (Rouault et al. 2000; Gibbs et al. 2003; Foley and Telonis–Scott 2010). A few studies compare both sexual and environmental selection on CHCs (Skroblin and Blows 2006).

**Figure 1:**
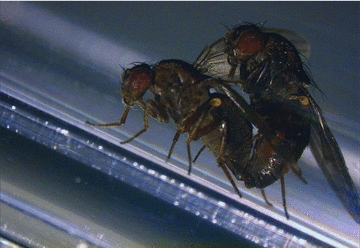
*Drosophila montana* mating pair (courtesy of Anne Lehtovaara).

*Drosophila montana* (Fig. 1) is a convenient species for investigating and characterizing sexual selection on male traits. Courtship song is almost obligatory for mating (Hoikkala 1988). Song carrier frequency (FRE) has been shown to be the target of female choice in laboratory and field populations from Finland (Aspi and Hoikkala 1995; Ritchie et al. 1998). It correlates with offspring survival in a Finnish population (Hoikkala et al. 1998) and has diverged between geographically remote populations (Klappert et al. 2007). Song is also known to contribute to sexual isolation between species: interpulse interval (IPI) is a character used by females to avoid heterospecific matings (Saarikettu et al. 2005). Less is known about CHC variation and behavior in *D. montana.* There are no qualitative (sex specific) differences in CHCs between the sexes, and only limited quantitative differences (Bartelt et al. 1986; Jackson and Bartelt 1986; Suvanto et al. 2000).

The presence of variation in the form, targets, or strength of sexual selection within a species is a necessary requirement for sexual selection to be a contributor to sexual isolation, and eventually speciation. In this study, we used two allopatric populations from distinct lineages of *D. montana*, from Finland (Oulanka) and Canada (Vancouver). They diverged about 0.5 million years (MY) ago (Mirol et al. 2007) and both pre- and postmating reproductive isolation have been observed between these populations (Jennings et al. 2011). We have analyzed the potential contribution of song and CHCs to courtship outcome both separately, and in combination. We present selection analysis under controlled conditions in the laboratory, on four independent song characters from the complete male courtship song bout before mating, and analyses of CHC profiles of males and females. The analysis used mating success, mating latency, and the production of rejection song by females (Satokangas et al. 1994) as fitness proxies. We find song to be a stronger predictor of courtship outcome than CHCs and we observe a relationship between sexual selection and population divergence. In particular, the greatest effects on courtship outcome involved the most divergent song characters, and selection on some traits was population specific, especially for CHCs.

## Materials and Methods

### Sampling

Isofemale lines were established from field collections made in June 2008 and 2009 from Vancouver, Canada (48°N, 123°W) (30 lines) and Oulanka, nd (66°N, 29°E) (42 lines), respectively. Within-population crosses were established for two (Oulanka) or three (Vancouver) generations and were scored for courtship song and CHCs.

### Fly rearing and mating trials

Flies were raised on a malt medium and kept in constant light at 19–20°C, which is necessary with this species to avoid diapause. Virginity was ensured by collecting newly emerged adults every 2–3 days and keeping them in vials separated by sex. All mating trials were conducted when flies were sexually mature (mean 21.1 days post eclosion, SD 5.2), at a mean temperature of 15.14°C (SD 1.51). Fly age did not influence any song component, or courtship outcome, while temperature had a strong effect on song (Table S1) and was added as a covariate in all models.

For the mating trials, one fly of each sex was introduced to a cylindrical plastic mating chamber (2 cm in diameter and 1.3 cm in height) and they were allowed 10 min to mate. Typically, a male would sing within 90 sec and there would be mating about 1 min after song production. Three measures of courtship outcome were recorded as proxies of sexual selection: mating within 10 min (binomial), rejection song production by females (binomial), and mating latency (time from first song production to mounting in seconds).

### Song analysis

**Figure 2:**
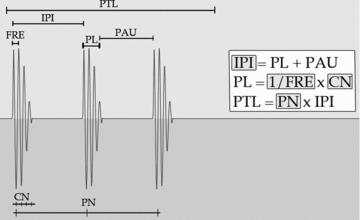
Illustration of song characters measured in the literature and their relationships. The top lines indicate durations, the bottom lines indicate counts. We used the four song characters indicated by squares for all analyses because they are independent. FRE = carrier frequency, IPI = interpulse interval, PL = pulse length, PAU = pause, CN = cycle number, PTL = pulse train length, PN = pulse number.

Male song was recorded with custom-made “Insectavox” microphones (Gorczyca and Hall 1987), within which the mating chambers were placed. Recordings were made directly to a computer as.wav files, after being band-pass filtered between 200 Hz and 2000 Hz. The.wav files were imported to Spike2 version 7 (Cambridge Electronic Design, Belmont, MA). We initially measured seven song traits but found strong covariation among some of them. Therefore, we report analyses of four traits that together are sufficient to describe song variation (Fig. 2). These were carrier frequency (FRE), interpulse interval (IPI), cycle number per pulse (CN), and pulse number per pulse train (PN). We used the average over the whole song (mean pulse trains 16.76, SD 18.9) produced by a male during a mating trial.

### CHC extraction and analysis

**Figure 3:**
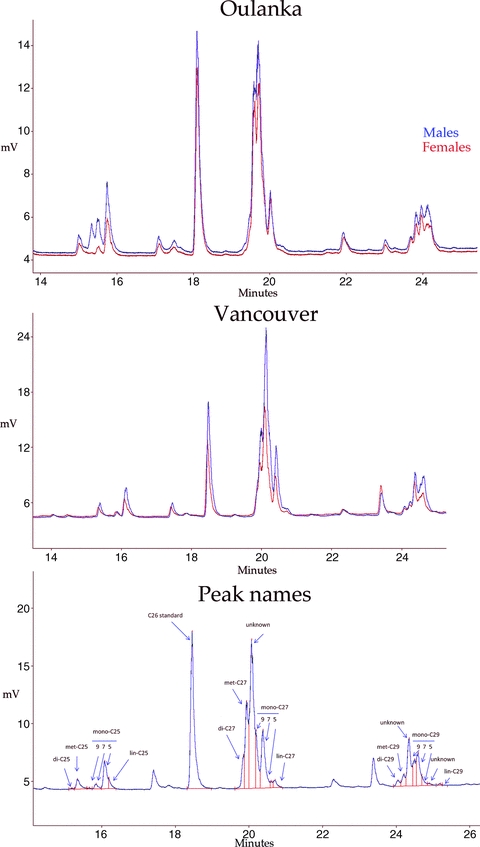
Examples of cuticular hydrocarbon (CHC) profiles for each population and sex. The named peaks were used for the calculations as they are hydrocarbons. The peaks that are not named correspond to even number hydrocarbons or nonhydrocarbons and were excluded from the analyses.

CHC components can vary due to social experience, including mating trials with females (Petfield et al. 2005; Kent et al. 2008; Etges et al. 2009; Everaerts et al. 2010). In order to minimize any direct influence of the mating trial, CHC extractions were made a considerable time after the trials (mean age 41 days, SD 6.7). This should not affect sexual maturity as *D. montana* overwinter as adults and reproduce over many months (Aspi et al. 1993). Flies were kept on normal food in single mating pairs with the same partner as the mating trial until extraction. During this period, the flies presumably experienced multiple matings, which should make them more comparable than they would have been straight after the trial. For extraction, CO_2_ anesthetized flies that had been kept at –20°C for at least 30 min were individually dipped in 400-µl heptane, containing 500-ng hexacosane (nC_26_H_54_) as an internal standard (Atchison 1986). The extraction time was 10 min, after which the flies were removed from the tube and the sample was left to evaporate. The vials were kept at –20°C until analyzed. The extracts were redissolved in 100-µl heptane for analysis by gas chromatography. A Perichrom gas chromatograph with a flame-ionization detector, equipped with a BP1 capillary column (25 m, 0.22 mm i.d., SGE Analytical Science Scientific Glass Engineering), with hydrogen as gas vector, was used. The oven temperature was programed from 180°C to 320°C at 3°C/min. PR2100 Perichrom Gas Chromatograph were acquired and analyzed with the WiniLabIII/Azur software. Eighteen peaks were named based on their retention time and their consistent presence in all individuals (Fig. 3).

The CHC data were transformed as in Rundle et al. (2009), to generate metrics of relative proportions of CHCs. Briefly, each CHC peak area was converted to the proportion of total CHC present, then divided by the proportion of one CHC (C_29:9_). Some CHCs had zero values in some individuals, which were replaced with a value 10× smaller than the minimum for the particular CHC over all individuals, to allow log transformation. All CHC data were log transformed and analyzed by principal component analysis in R version 2.10.1 (R Development Core Team 2007). Finally, the principal components were transformed to have a total variance of 1 (by dividing with the square root of the sum of the variance of all the scores) to make the principal components from song characters and CHCs directly comparable.

### Selection analysis

For all analyses, the data for the two populations were pooled. The binomial traits (mating and rejection song) were analyzed as in Fairbairn and Preziosi (1996), that is, *P*-values were obtained from generalized linear mixed models with binomial errors while regression coefficients were obtained from models of the binomial traits expressed relative to their averages. Models for mating latency were calculated with the lmer command of the lme4 library of R version 2.10.1 (R Development Core Team 2007). The *P*-values for mating latency were obtained by sampling 100,000 Markov chain Monte Carlo simulations based on the model, using the pvals.fnc command of the languageR library of R. The *P*-values for the binomial models were taken from the lmer output. The strength of quadratic selection reported is twice the coefficient for the squared term in each model (Stinchcombe et al. 2008).

For all models, generation was fitted as a random variable. Independent variables included temperature, population, and other song and CHC variables. The models included both linear and quadratic terms for the song and CHC variables, and their interactions with temperature (song only) and population. Initial models were simplified by dropping the interactions of the quadratic terms first and then, if possible, the linear terms, as long as doing so did not significantly increase model deviance.

All analyses were based on multivariate models, which measure selection acting on each trait after having taken into account variation in other traits. In total, there were three types of selection analysis: (1) Song selection analysis, which used the four song characters (FRE, IPI, CN, PN) after normalization, to make their effect sizes directly comparable. (2) CHC selection analysis, which used principal components calculated from both sexes combined. Models were fitted separately to the male and female data. (3) Combined selection analysis on both song and CHC data, to estimate their relative importance. These used principal components of the four song characters along with the previously calculated principal components from CHC data. The analysis was performed on males only, since females do not produce courtship song. Only principal components that explained >5% of the variance were used in all cases.

## Results

### Traits and population differences

In total, there are data from 909 individuals, of which 448 were males. The populations differed in all song traits. The greatest difference was in FRE, confirming the result of Klappert et al. (2007), and then in IPI, CN, and PN (Table 1 and Table S1). Higher temperature increased FRE and reduced all other characters, resulting in overall faster song.

**Table 1:** Means and standard errors of the song data from the two populations. The song characters have been corrected for the median temperature of 15.5°C. The populations significantly differ in all four traits (Table S1)

	**Vancouver**	**Oulanka**
**FRE (Hz)**	233 ± 37	266 ± 30
**IPI (msec)**	43.438 ± 6.952	40.2 ± 6.783
**PN**	4.821 ± 0.891	5.155 ± 1.165
**CN**	10.206 ± 1.341	9.263 ± 1.304

FRE = carrier frequency, IPI = interpulse interval, PN = pulse number, CN = cycle number.

Occasionally (in about 20% of the trials in either population), female flies produced a sound, which significantly reduced the probability of mating and increased mating latency (Table 2). The results are consistent with an interpretation as rejection song (Satokangas et al. 1994). Both the probability of mating and mating latency differed between populations, even after accounting for the incidence of rejection song (Table 2).

**Table 2:** Summary of the effects of rejection song on mating (χ^2^-test) and its latency (Spearman's statistic). Rejection song influenced both measures of mating success

Population		Rejection	No rejection	χ^2^–*r*	*P*-value
**Both**	**Mating**	47	370	62.79	**<0.001**
	**No mating**	74	113		
	**Latency**	NA		0.206	**<0.001**
**Vancouver**	**Mating**	19	192	29.42	**<0.001**
	**No mating**	39	77		
	**Latency**	NA		0.239	**<0.001**
**Oulanka**	**Mating**	28	178	36.30	**<0.001**
	**No mating**	35	36		
	**Latency**	NA		0.211	**0.0023**

**Figure 4:**
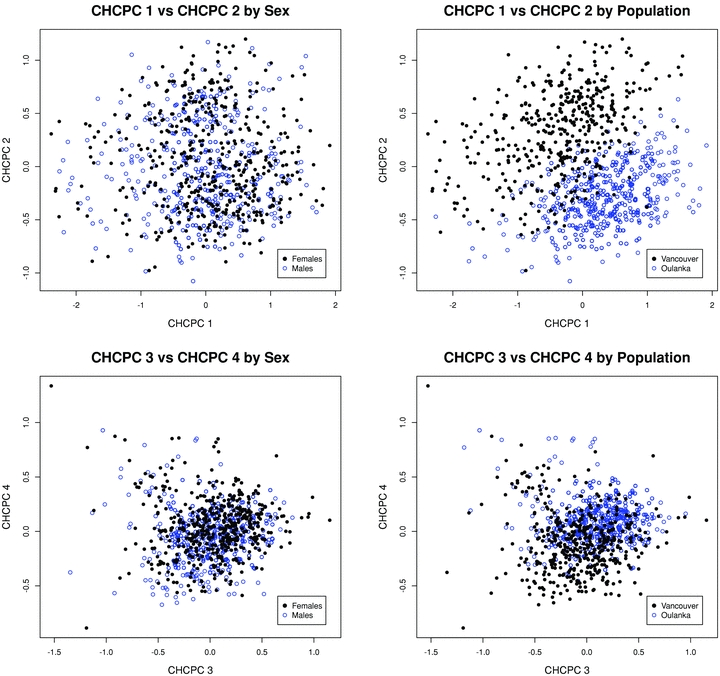
The CHC principal components (CHCPCs) used in selection analyses, distinguished by population or sex. Sexual dimorphism is limited and of less magnitude than population differentiation.

The proportions of variance explained by the first three song principal components (SongPCs) were 47%, 31%, and 17% (Table 3). SongPC 1 indicated fast song overall, with FRE and IPI having the greatest, roughly equal and inverse, influence. The remaining SongPCs were affected by different combinations of song characters (Table 3). The proportions of variance explained by the first four CHC principal components (CHCPCs) were 44%, 16%, 9%, and 6% (Table 4). Overall, CHC differences between populations were considerably greater than differences between the sexes (Fig. 4; Table 5).

**Table 3:** Loadings of the song principal components

Variable	SongPC 1	SongPC 2	SongPC 3
FRE	0.647	−0.210	−0.340
IPI	−0.666	−0.209	0.184
PN	−0.243	−0.717	−0.583
CN	−0.281	0.631	−0.715
SD	1.374	1.112	0.836
Variance	47.2%	30.9%	17.4%

**Table 4:** Loadings of the cuticular hydrocarbon (CHC) principal components. Values greater than 0.1 are indicated in bold

	CHCPC 1	CHCPC 2	CHCPC 3	CHCPC 4
C_25.di_	**−0.159**	**0.386**	**−0.174**	**−0.391**
C_25.met_	**−0.303**	**−0.191**	**−0.161**	**−0.162**
C_25.9_	0.031	**−0.486**	**−0.223**	**−0.236**
C_25.7_	**−0.204**	**−0.443**	**−0.175**	**−0.166**
C_25.5_	**−0.321**	−0.059	0.098	**0.136**
C_25.lin_	**−0.150**	**−0.356**	**0.364**	0.068
C_27.di_	−0.002	**−0.353**	**0.360**	**0.271**
C_27.met_	**−0.318**	−0.092	**−0.144**	0.004
C_27.9_	**−0.301**	0.077	0.066	0.063
C_27.7_	**−0.335**	**0.141**	0.075	−0.018
C_27.5_	**−0.254**	**0.177**	**0.305**	**0.240**
C_27.lin_	**−0.256**	**0.185**	**0.242**	**−0.260**
C_29.di_	**−0.331**	0.074	0.005	**−0.155**
C_29.met_	**−0.309**	−0.014	**0.125**	**0.102**
C_29.7_	**0.205**	**0.106**	**0.328**	0.076
C_29.5_	**0.164**	−0.021	**0.373**	**−0.376**
C_29.lin_	0.084	**−0.105**	**0.384**	**−0.575**
SD	**2.744**	**1.655**	**1.23**	**0.978**
Variance%	**44.3**%	**16.1**%	**8.9**%	**5.6**%

**Table 5:** Population and sex effects on CHC principal components (CHCPCs) estimated from linear mixed models. Population indicates the effect of Vancouver relative to Oulanka and Sex the effect of Males relative to females. The *P*-values were obtained from mcmc simulations

Variable	Population	Sex	Population: Sex
CHCPC 1	−0.919***	−0.140**	NA
CHCPC 2	0.625***	−0.128***	NA
CHCPC 3	−0.153***	−0.0013	−0.088*
CHCPC 4	−0.155***	−0.045	−0.070*

Significance level

* <0.05

** <0.01

*** <0.001.

NA = dropped from model.

### Song selection analysis

**Figure 5:**
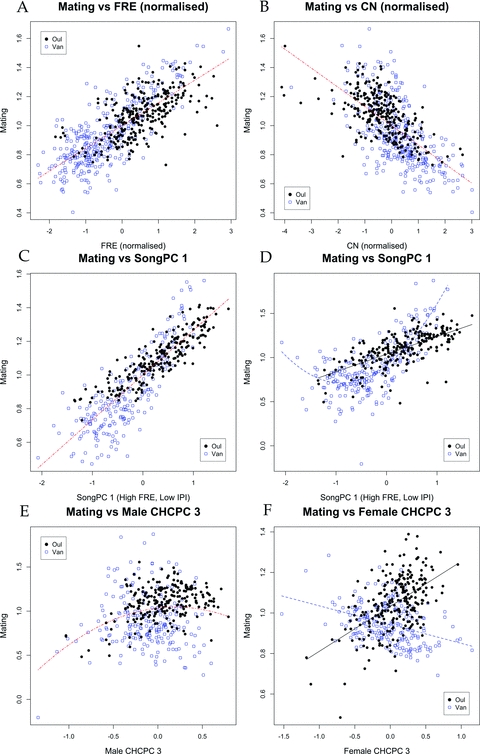
Fitted relationships for the significant predictors of mating success within 10 min. The points are predicted values based on other terms in the model. All plots of principal components are based on the combined song and CHC models, except (C), which is based on a model with song principal components (SongPCs) only.

The results of selection analysis on song characters are summarised in Table 6. In general, different song components influenced each measure of courtship outcome. Faster song (high FRE, low CN, low IPI, Figure 5A-D) was associated with higher mating success, in agreement with previous work (Aspi and Hoikkala, 1995, Hoikkala *et al*., 1998, Klappert *et al*., 2007).

**Table 6:** Summary of the significant partial selection coefficients from the song selection analysis models. Population indicates the effect of Oulanka relative to Vancouver. Interactions are indicated by colons and quadratic effects by “^2^.”

	Courtship outcome		
	
Variable	Mating	Mating latency	Rejection song
Temperature	−0.055^**^	0.121^**^	0.151*
Population	NS	NS	0.704^**^
FRE	0.218^***^	NS	NS
CN	−0.092*	NS	NS
IPI	NS	0.467^**^	NS
IPI^2^	NA	−0.342^***^	NS
Population: PN	NS	−0.283*	0.455*
Population: PN^2^	NA	NA	−0.498^**^

Significance level: * <0.05, ^**^ <0.01, ^***^ <0.001.

NS = not significant, NA = dropped from model.

**Figure 6:**
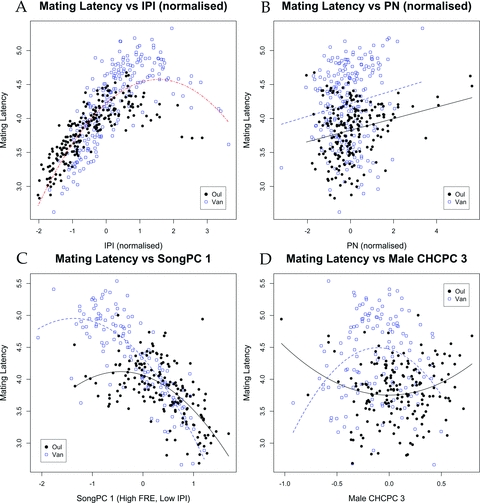
Fitted relationships for the significant predictors of mating latency. The points are predicted values based on other terms in the model. All plots of principal components are based on the combined song and CHC models.

Selection coefficients were mostly consistent between the two populations. Those based on mating latency showed both directional and quadratic (disruptive) components of selection on IPI (Fig. 6A and 6C) plus differing directional selection on PN between populations (Fig. 6B). Shorter IPI and lower PN led to faster mating, with the directional effect dominating over the disruptive component over most of the phenotypic range of IPI. Lower temperatures led to higher fitness (shorter mating latency, more mating, and less rejection song), which might be expected since *D. montana* is a cold-adapted species.

**Figure 7:**
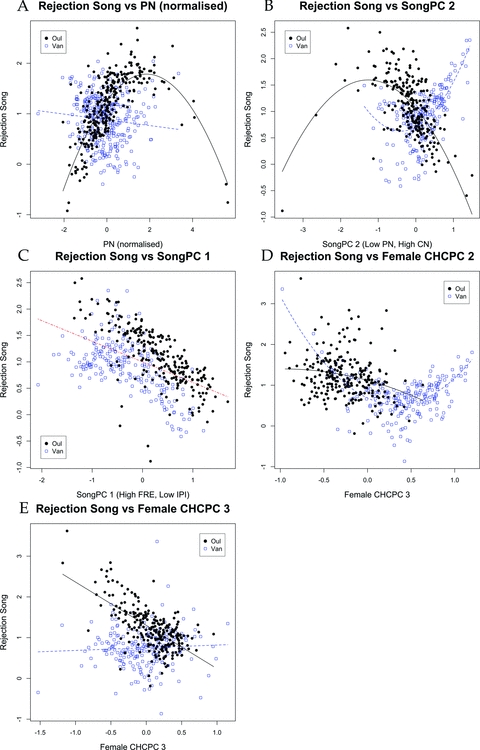
Fitted relationships for the significant predictors of female rejection song. The points are predicted values based on other terms in the model. All plots of principal components are based on the combined song and CHC models.

PN was the strongest predictor of rejection song, but the nature of the selection (both linear and quadratic) differed greatly between populations (Fig. 7A and 7B). PN had little influence on the production of rejection song in Vancouver, while in Oulanka, high PN increased rejection song over most of the parameter space, while extremely high PN produced by a few individuals reduced it, resulting in disruptive selection overall.

### CHC selection analysis

CHC selection analyses are summarized in Table 7. The three courtship outcomes were primarily influenced by one CHCPC (CHCPC 3) in both sexes (Figs. 5E and 5F, 6D, and 7E). CHCPC 2 additionally influenced rejection song differently in the two populations (Fig. 7D). Overall, male and female CHCPC effects were limited but included strong linear selection toward opposite directions in the two populations (Figs. 5F and 7E), which is the form of selection most likely to contribute to population divergence.

**Table 7:** Summary of CHC selection analysis. Only the significant partial selection coefficients, based on CHC components normalized for total variance explained, are shown. Population indicates the effect of Vancouver relative to Oulanka. Interactions are indicated by colons and quadratic effects by “^2^.”

		Courtship outcome		
		
	Variable	Mating	Mating latency	Rejection song
Females	CHCPC 3	0.379*	NS	−1.603^**^
	Population: (CHCPC 3)	–0.539*	NS	1.916*
	Population: (CHCPC 2)^2^	NA	NA	5.322*
Males	(CHCPC 3)^2^	−1.113*	NS	NA
	Population: (CHCPC 3)^2^	NA	–5.744*	NA

Significance level: * <0.05, ^**^ <0.01, ^***^<0.001.

NS = not significant, NA = dropped from model.

### Song and CHCs

Results from selection analysis on song and male CHCs combined are summarized in Table 8. The general trend was that there were fewer CHCPC effects compared to song effects, for example, no male CHCPC predicted rejection song, but the CHCPC effects were of greater magnitude. The most interesting song effects were those that remained significant after accounting for CHCPC variation. They were FRE and IPI since SongPC 1, largely reflecting “fast” song (high FRE, low IPI, Table 3), significantly predicted all courtship outcomes. In addition, SongPC 2 (high CN, low PN, Table 3) predicted the probability of rejection song. The CHCPC effects remained the same after accounting for song variation but increased in significance.

**Table 8:** Summary of combined song and CHC analysis. The numbers indicate effect sizes, normalized for total variance explained by all (song or CHC) principal components used. Population indicates the effect of Vancouver. Interactions are indicated by colons and quadratic effects by “^2^.”

	Courtship outcome		
	
Variable	Mating	Mating latency	Rejection song
Temperature	−0.054*	0.140^**^	0.149*
SongPC 1	0.259^**^	−0.377*	−0.770^**^
SongPC 2	NS	NS	−0.693*
(SongPC 1)^2^	NS	−0.530*	NS
(SongPC 2)^2^	NS	NS	−0.747*
(CHCPC 3)^2^	−1.114^**^	NS	NS
Population: (SongPC 1)	NS	−1.358^**^	NS
Population: (SongPC 2)	NS	NS	0.969*
Population: (SongPC 1)^2^	0.487*	NS	NS
Population: (SongPC 2)^2^	NS	NS	1.612*
Population: (CHCPC 3)^2^	NS	−6.359^**^	NS

Significance level: * <0.05, ^**^ <0.01, ^***^ <0.001.

NS = not significant.

Overall, song explained more of the variance in courtship outcome than either male or female CHCs, with the exception of the production of rejection song, which was equally well explained by variation in song and female CHCs (Table 9). Comparison of the variance explained by each type of model reveals that song and male CHCs acted in an additive manner (Table 9).

**Table 9:** Comparison of song and CHC models. The numbers indicate adjusted *R*^2^ values from regressions between the fitted values of selection analysis models against the relevant courtship outcome

	Courtship outcome		
	
Variables	Mating	Mating latency	Rejection song
Male CHCPCs	0.06	0.10	0.03
Female CHCPCs	0.04	0.04	0.08
Song and male CHCPCs	0.16	0.27	0.11
Song characters	0.09	0.21	0.08

## Discussion

Here, we have analyzed the associations between song and CHC variation with measures of courtship outcome in two distinct natural populations of *D. montana*. The measures were the incidence of mating, mating latency, and the incidence of rejection song by females. We first discuss the interplay between sexual selection on different traits and their relative contribution to mating success in *D. montana*. We then comment on the potential of sexual selection to promote divergence between populations given the similarities in traits under selection and the direction of divergence found between the populations.

### Mate choice in D. montana

Both song and CHCs were found to be significant predictors of mating success. The variation explained by song was usually greater than that explained by CHCs. Still, CHCs clearly predicted mating success despite being qualitatively sexually monomorphic, in contrast to the expectations of Bartelt et al. (1986). In other *Drosophila* species, CHCs seem to have a more prominent role. For example, in hybrids of *D. serrata* and *D. birchii*, the average genetic correlation between mating success and CHC profile was 0.84 (Blows and Allan 1998). In this case, CHCs explained most of the variance in mate choice, however song was not analyzed.

Few studies have compared sexual selection on song and CHCs of *Drosophila* combined. In *D. mojavensis*, the significance of CHCPCs dropped markedly when song was taken into account (Etges et al. 2009), implying that CHCs and song covary in their effects. In other studies, the effects seem additive and are usually not equal. In *D. melanogaster*, Rybak et al. (2002) experimentally removed either courtship song or CHCs from males and showed a greater reduction in mating success when song was absent, which led to the conclusion that the traits acted synergistically. Similarly, in an experiment controlling signal perception, rather than the signal itself, there was a greater reduction in mating of deaf flies compared to olfaction-deficient ones (Markow 1987). Regardless of the relative importance of different courtship signals, cases where both songs and CHCs are studied show that their combination always explains, or results in, more mating success (Rybak et al. 2002; Etges et al. 2009) supporting a multimodal nature of animal communication.

It is particularly important to study both signal modalities if they interact. In *D. montana*, song and CHCs had similar and independent effects, since the variation explained by the combined model was approximately equal to the sum of the variation explained in the models with either signal (Table 9). One interesting pattern was the relationship between SongPC 1 and mating incidence, which differed between populations only when CHC variation was included in the model (compare Fig. 5C and 5D). There were two other cases in which the fitness surface showed differences between the populations in the combined model compared to the models of only one signal modality. They involved the relationship between IPI and mating latency and that between PN and rejection song (Figs. 6A and 6C and 7A and 7B, respectively). In all three cases, the differences between the populations became more pronounced.

Interestingly, female CHCPCs explained more variation in rejection song than male CHCPCs (Table 9). The data are compatible with a role of some female CHCs as pheromones that induce courtship from males, as in *D. melanogaster* (Jallon 1984). Female CHCs would then induce more rejection song compared to male CHCs because males would court attractive females more. Attractive females would thus produce rejection song more often than unattractive ones. An alternative possibility is that different female CHC profiles could trigger different male courtship, which, in turn, could lead to female rejection behavior.

### Mate choice is a response to multiple signals

One striking result was that different traits influenced the different measures of courtship outcome (i.e., Tables 6, 7, and 8 have different significant variables in each column). Yet, those measures are not independent: mating latency involves a subset of flies from the mating trials, and both mating incidence and latency were below average when rejection song was heard (Table 2). The fact that there was no complete overlap between the traits influencing the three measures of mating success suggests that mate choice is the outcome of independent choices perceived though different sensory modalities, such as auditory or olfaction/gustation, which sum to influence the probability of mating.

Rejection song has been described before in *D. montana* (Satokangas et al. 1994). It is common in young females or those courted by heterospecific males and inhibits male courtship attempts (Liimatainen and Hoikkala 1998). It may function to avoid heterospecific matings or to prolong courtship, perhaps in order to test a male further, or to sample more males. The interpretation of rejection song is difficult in this study because the confined space of our experimental setup may have caused females to produce it, while in more natural conditions, they could simply fly away.

### Comparison of the results with previous studies

The consistency between the populations in the direction of sexual selection on song is in agreement with its potential function as an honest indicator of fitness, as previously suggested for *D. montana*. Strong directional selection on FRE has been previously observed (Hoikkala 1988; Hoikkala and Aspi 1993; Ritchie et al. 1998), though the importance of other pulse traits varies between studies. Playback experiments using artificial song (Ritchie et al. 1998, 2001) found high FRE and short pulse length (PL) to be attractive, which is compatible with the selection on song found here (Fig. 2). In addition, studies of song variation in Finnish *D. montana* have suggested that “fast” song, which was captured by SongPC 1 in this study, may be an indicator of male quality: fast song predicts offspring quality (Hoikkala et al. 1998), is sensitive to environmental conditions (Hoikkala and Isoherranen 1997), and declines with age (Hoikkala et al. 2008). It also shows directional dominance, which is consistent with strongly directional selection (Suvanto et al. 2000). If high FRE song is a relatively simple condition-dependent signal, patterns of sexual selection may be consistently directional between populations.

Courtship, mating, and social experience can have confounding effects on CHC expression (Petfield et al. 2005; Kent et al. 2008; Krupp et al. 2008; Etges et al. 2009; Everaerts et al. 2010). We scored the CHCs about 20 days after the mating trial in order to avoid such short-term effects. However, this also means the CHCs scores were not necessarily an accurate reflection of what the flies encountered during a mating trial. While age did not seem to affect CHCs (it could be dropped from all models), it is possible that mating status of the flies did. Possible remating of the flies may have minimized the differences between the sexes but it would make the flies more comparable than if they had been scored immediately after the trial when some had not mated. No qualitative differences were found between the sexes even when they had been kept separately after mating (J. H. Jennings unpublished). While a lag between mating and CHC scoring may confound our interpretation, it should be conservative, and the fact that we find significant associations between mating and CHCs imply that CHC variation between individuals is an important component of mating success in *D. montana*.

### Sexual selection as a force of population divergence

Population differentiation on CHCs in *D. montana* has been reported before (Suvanto et al. 2000) but some populations were based on lines maintained in the lab for 20 generations, which may have altered their CHC profiles. In this study, population differences were greater than sexual dimorphism (Fig. 4; Table 5). CHCPC 3, which consistently affected mating success and sometimes showed opposite linear selection in the two populations, showed a weak interaction between population and sex and was significantly divergent between the populations in a pattern consistent with the variation in selection (Table 5). Our data thus provide some support for sexual selection on CHCs leading to population differentiation along this axis, however abiotic environmental variation may also have contributed to population differentiation in CHCs, for example long CHCs are associated with desiccation tolerance in insects (Howard and Blomquist 1982).

The divergence between the populations was not always consistent with the patterns of sexual selection found here. The populations differed in song characters, in particular, despite them experiencing similar sexual selection. One possible explanation is if the sexually selected traits were condition dependent, but the optimal conditions differed between populations (van Doorn et al. 2009). Another possibility would be a stronger opposition by natural selection in one population. Vancouver and Oulanka differ in many ways, including photoperiod, altitude, and number of generations in a year (Jennings et al. 2011), though it is impossible to understand how climatological or other variables may influence song and CHC expression with the current level of population sampling. A third possibility is reproductive character displacement due to sympatry with closely related species, which may lead to divergence away from the optimal phenotype under sexual selection (see Higgie and Blows (2008) for an example with *Drosophila* CHCs). The population from Oulanka is sympatric with the closely related *D. littoralis* and *D. ezoana*, while the population from Vancouver largely exists in allopatry from other virilis group species (Liimatainen and Hoikkala 1998, pers. obs.). Overall, the balance between sexual and ecological selection may be more important than the nature of sexual selection alone in predicting population differences (Ritchie 2007; Kraaijeveld et al. 2010).

### Sexual selection and speciation

The most novel target of sexual selection found here was IPI, detected directly in the song model of mating latency, and in the combined models, all of which showed significant SongPC 1 effects. SongPC 1 was heavily influenced by both FRE and IPI, however its IPI component is not simply due to covariation with FRE: The characters correlate strongly (≍0.68) in both populations of this study (Table 10) but when these populations were crossed the characters did not correlate in the F_2_ and independent QTLs explained variation in FRE and IPI (Lagisz et al. in press), so any genetic correlation is not due to pleiotropy. The strong effect of IPI on mating success, found in this study, may therefore be due to selection on IPI itself. IPI is the song character most commonly diverged between closely related species in the virilis (Hoikkala et al. 1982; Hoikkala and Lumme 1987) and other groups of *Drosophila* (Ritchie and Gleason 1995; Ritchie et al. 1999). Our data are the first to show sexual selection on IPI in *D. montana*, thus providing a potential link between sexual selection and speciation.

**Table 10:** Song character correlations. The characters have been corrected for the median temperature (15.5°C). Spearman *r* values are shown on the bottom left triangle, *P*-values on the top right and are indicated in bold when significant

			**FRE**	**CN**	**PN**	**IPI**
**Population**	**Oulanka**	**FRE**	-	**0.011**	0.**007**	**<0.001**
		**CN**	–0.153	–	0.060	0.284
		**PN**	–0.160	0.113	–	**<0.001**
		**IPI**	–0.606	0.065	0.537	–
	**Vancouver**	**FRE**	−	**<0.001**	0.276	**<0.001**
		**CN**	–0.225	–	**0.046**	0.083
		**PN**	0.0604	–0.110	–	**<0.001**
		**IPI**	–0.73	0.096	0.175	–
	**Both**	**FRE**	−	**<0.001**	0.078	**<0.001**
		**CN**	–0.346	–	0.252	**<0.001**
		**PN**	0.072	–0.047	–	**<0.001**
		**IPI**	–0.687	0.165	0.291	–

## Conclusion

We have examined the complex interplay between mating success and variation in both song and CHC components in two natural populations of *D. montana*. The fitted selection surfaces are complex, with different predictors of mating success implying that different forms of selection operate on these traits. Both traits correlate with mating success, and seem to do so in an additive manner. In general, selection is consistent between the populations over much of the observed variation in traits. Curiously, while the traits under strongest selection differ most between populations, the selection surface implies that selection acts consistently in both populations, and traits that show differing selection surfaces vary less between populations. These results emphasize that studies of sexual selection alone cannot predict differences between populations.
